# Replica-mold nanopatterned PHEMA hydrogel surfaces for ophthalmic applications

**DOI:** 10.1038/s41598-022-18564-3

**Published:** 2022-08-25

**Authors:** Tomáš Krajňák, Eva Černá, Markéta Šuráňová, Tomáš Šamořil, Daniel Zicha, Lucy Vojtová, Jan Čechal

**Affiliations:** 1 CEITEC – Central European Institute of Technology, Brno University of Technology, Purkyňova 123, 612 00 Brno, Czech Republic; 2 Institute of Physical Engineering, Brno University of Technology, Technická 2896/2, 616 69 Brno, Czech Republic

**Keywords:** Materials science, Nanoscience and technology

## Abstract

Biomimicking native tissues and organs require the development of advanced hydrogels. The patterning of hydrogel surfaces may enhance the cellular functionality and therapeutic efficacy of implants. For example, nanopatterning of the intraocular lens (IOL) surface can suppress the upregulation of cytoskeleton proteins (actin and actinin) within the cells in contact with the IOL surface and, hence, prevent secondary cataracts causing blurry or opaque vision. Here we introduce a fast and efficient method for fabricating arrays consisting of millions of individual nanostructures on the hydrogel surface. In particular, we have prepared the randomly distributed nanopillars on poly(2-hydroxyethyl methacrylate) hydrogel using replica molding and show that the number, shape, and arrangement of nanostructures are fully adjustable. Characterization by atomic force microscopy revealed that all nanopillars were of similar shape, narrow size distribution, and without significant defects. In imprint lithography, choosing the appropriate hydrogel composition is critical. As hydrogels with imprinted nanostructures mimic the natural cell environment, they can find applications in fundamental cell biology research, e.g., they can tune cell attachment and inhibit or promote cell clustering by a specific arrangement of protrusive nanostructures on the hydrogel surface.

## Introduction

Hydrogel materials composed of hydrophilic crosslinked polymeric chains are widely used in tissue engineering due to their ability to absorb and retain a large amount of water while maintaining their insoluble three-dimensional network structure^1^. The biocompatibility of hydrogels has been extensively investigated with respect to their use in the biomedical industry since the pioneering work of Wichterle and Lim in 1960^2^. Hydrogels from natural polymers provide several advantages, such as biocompatibility, cell-controlled degradation, and intrinsic cellular interaction^3^. However, they have a limited range of mechanical properties. In contrast, synthetic polymers can be prepared with precisely controlled structures and functions, although their limited degradability in physiological conditions and toxic chemicals may present drawbacks^4^. One of the most studied synthetic hydrogels is based on poly (2-hydroxyethyl methacrylate, PHEMA). This polymer is synthesized from the precursor monomer 2-hydroxyethyl methacrylate (HEMA) via thermally or radiatively (gamma, UV, blue-light) initiated free radical polymerization^5^. PHEMA is a transparent, biocompatible, nontoxic, non-degradable, non-adhesive, hydrophilic hydrogel material with a high and tunable mechanical strength^6^, and is applied in the field of biomedicine, particularly in ophthalmology, especially as contact and intraocular lenses^7^.

Many advances and modifications of hydrogels have been developed throughout the years that utilize the PHEMA hydrogel for various biomedical applications^8^. One of the essential areas of application is drug, protein, or cell delivery for tissue engineering^9^. PHEMA can be directly injected into the body, and is minimally invasive^10^. The drug is usually encapsulated inside a hydrogel^11^. The microporosity of the hydrogel polymers can also be used to control the diffusion of molecular species into and out of the hydrogel interior. For example, adjustment of the mesh size of the polymer can result in the size-selective exclusion of proteins and other unwanted contaminants in therapeutic and diagnostic applications^12^. Another prominent application of hydrogels is incorporating growth factors (vascular endothelial, basic fibroblast, epidermal, and bone morphogenetic protein) into PHEMA, promoting the formation of blood vessels^13^. Nanostructuring of the surface would provide a range of new application possibilities, e.g., suppressing the formation of cytoskeleton proteins^14^ on the surface of the intraocular lens (IOL), which could induce its change from transparent to opaque. Confirming this hypothesis will be essential in producing an IOL resistant to secondary cataracts. However, it is necessary to develop scalable methodologies to produce large surface areas with low costs to enable these applications.

Lithography methods combined with replica molding (also called cast molding) allow imprinting of the patterned structure from a solid mold to a hydrogel surface during its preparation^15,16^; the negative and positive features on the mold results in protrusions or pores. Another modification method of hydrogel chemistry and surface topography can be achieved by excimer laser^17^. Laser modification treatment allows changes in physicochemical surface properties, e.g., the laser-induced structures can control cytocompatibility or be applied as antibacterial substrates and in plasmonic-based detection systems^18^. The hydrogel protrusions can alter surface topography, which mimics the cell's in vivo environment and influences cell or protein attachment^19^. Such a modified surface allows for studying cell-material interactions^20^.

This paper reports nanostructured pattern fabrication on the PHEMA hydrogel consisting of randomly distributed nanopillars invisible to the human eye. We have employed a focused ion beam (FIB) to fabricate a stamp mold with the desired nanopillar array and transferred the pattern onto the hydrogel using replica molding. The employed fabrication method is fast and reproducible. We also discuss proper hydrogel blend compositions for printing and describe nanopillar dimensions for different patterns, and we have applied a broad set of analytical techniques to show that nanostructured hydrogel retains its favorable properties for ophthalmic applications. This study extends the possible applications of modified hydrogels, which could be used in ophthalmology as cataract-resistant intraocular lenses.

## Materials and methods

### Stamp mold

As a mold, we have selected a piece of silicon (1 × 1 cm^2^) cut from a (100) oriented boron-doped silicon wafer comprising a 1–2 nm-thick native oxide layer (Sil'tronix company). The silicon molds were fabricated using a focused ion beam in the LYRA3 FIB-SEM instrument (TESCAN), using 30 keV gallium ions (beam currents 160 pA and 42 pA). The parameters of the mold pattern were defined by the custom script (see Supplementary Information for detailed script description). The silicon mold patterns were characterized by SEM within the same instrument using an accelerating voltage of 5 kV and a beam current of 160 pA.

### Hydrogel PHEMA and its nanopatterning

The poly(2-hydroxyethyl methacrylate) (PHEMA) hydrogel was prepared using photopolymerizing 2-hydroxyethyl methacrylate (HEMA) (purity ≥ 99%, Sigma-Aldrich). For polymerization, the ethylene glycol dimethacrylate (EGDMA) (purity 98%, Sigma-Aldrich) was used as a crosslinking agent, and the 2,2-dimethoxy-2-phenyl acetophenone (DMPA) as a photoinitiator (purity 99%, Acros Organics). The employed ultrapure water (type 1 according to ISO 3696) was prepared using a Millipore purification system (MilliQ Academic, Millipore, France).

### Characterization of PHEMA nanostructures

The morphology and dimensions of the milled features and imprinted patterns on hydrogel were measured using the Bruker Dimension Icon atomic force microscope (AFM) with a ScanAsyst tip in contact mode in the air (peak force setpoint 2 nN, feedback gain 15, scan size 5 × 5 μm^2^, 512 × 512 pixels). The measured AFM data were processed by common approaches implemented in the Gwyddion software^21^. The corrected images were used to determine the depth and height of the nanostructures directly in Gwyddion and their diameter using ImageJ software^22^.

X-ray photoelectron spectroscopy (XPS) of the dried hydrogels was measured using the Kratos AXIS Supra employing monochromatized Al Ka radiation (emission current 15 mA). The photoelectrons were collected in normal emission geometry with the magnetic lens turned on. Overview spectra were acquired with a pass energy of 80 eV with a 1 eV energy step. Detailed photoelectron spectra were measured employing a pass energy of 20 eV with a 0.1 eV energy step. An electron flood gun was used for charge neutralization; the charge-induced shift was corrected to the binding energy of 285.0 eV of the main C 1 s peak component^23^.

The Fourier-transform infrared spectroscopy (FTIR) was performed on dried PHEMA hydrogel samples within and outside the nanopattern using the Hyperion 3000/Vertex (Bruker). The reflectance was measured in a wavenumber range between 4000 and 600 cm^−1^ with 128 scans per sample and at a 4 cm^−1^ resolution in ambient air conditions.

The wide-angle X-ray scattering (WAXS) measurements were done on a Rigaku Smartlab 3 instrument equipped with a rotating Cu anode and parallel beam setup to produce Cu Kα radiation (λ = 0.1541 nm); the 0.3 mm pinhole and 0.2 mm collimator restrict the beam size to approximately 0.3 mm in diameter. Data were collected using a HyPiX-3000 active-matrix two-dimensional detector positioned at 150 mm from the sample. WAXS was measured in transmission geometry. Two-dimensional diffractograms were measured from each scanning area. The intensity from diffractograms was recalculated by azimuthal integration to obtain a one-dimensional radial profile. The spacing was determined from the Bragg law^24^, which uses identified peak positions from the profiles. The spectra from 0 to 10° were not included because they were influenced by a large error caused by air scattering.

The light transmissivity was studied in the bright-field mode using a Nikon Eclipse Ti microscope equipped with LU Plan Fluor 10 × NA 0.3 objective lens, white light halogen illuminator, Jenoptic Progres MF microscope camera, and NIS software (Nikon).

The water contact angle of the hydrogel surface was measured and evaluated by the See System E (Advex Instrument s.r.o.) using the software See System for Surface Energy Measurement. Water droplet (5 μl) were applied from the micro-syringe on dried and purified PHEMA hydrogel. A measurement was repeated twelve times to obtain enough statistics. Additional analysis of the dried PHEMA samples dedicated to the water contact angle can be found in Supplementary Information.

## Results

### Fabrication of stamp molds

**Figure 1:**
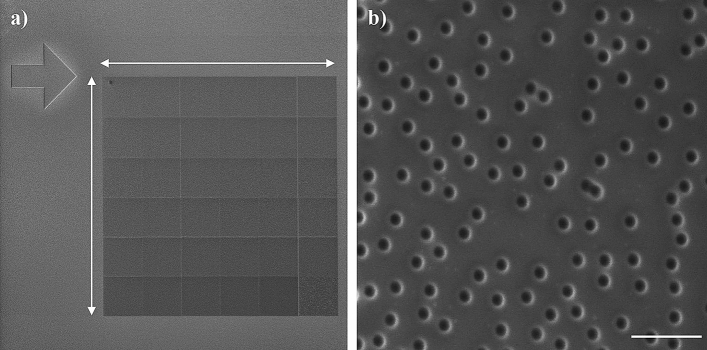
(**a**) Overview of the nanopatterned array with an area 540 × 540 µm^2^ consisting of 1,361,700 holes on the silicon mold. (**b**) The detailed view of the array of big (30 kV, 160 pA) holes (scale bar 1 µm). Images were acquired by SEM (5 kV, 160 pA).

**Figure 2:**
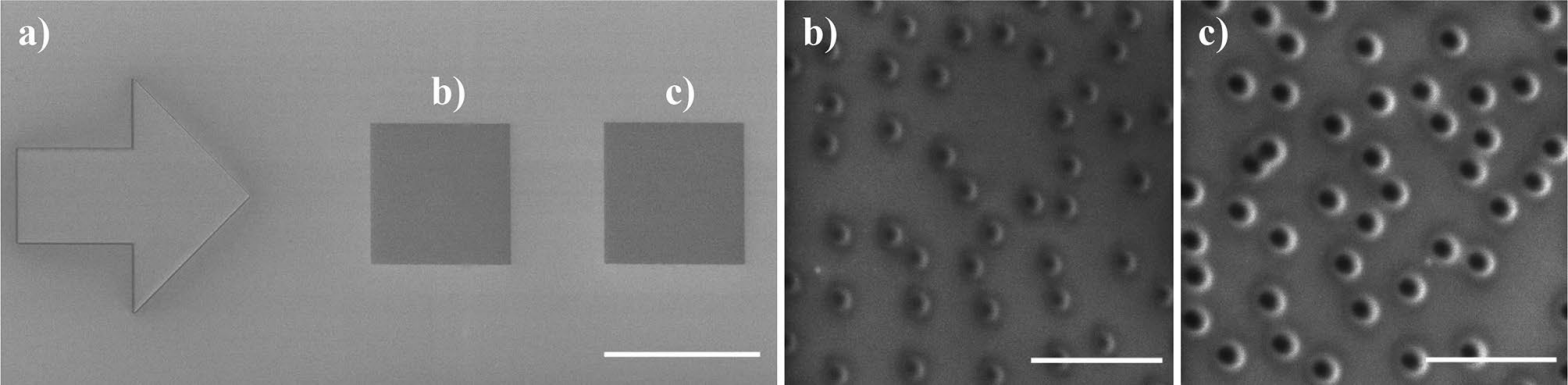
(**a**) Silicon mold with arrow-shaped marker and two 90 × 90 µm^2^ arrays, each consisting of 37,825 holes (scale bar 100 µm). (**b**,**c**) Detailed view of arrays of (**b**) small (30 kV, 42 pA) and (**b**) big (30 kV, 160 pA) holes (scale bars 1 µm). Images were acquired by SEM (5 kV, 160 pA).

This work aims to obtain a hydrogel surface featuring arrays of randomly distributed nanopillars of specific dimensions by replica molding. As a mold, we have employed a piece of silicon with a 1–2 nm-thick native oxide layer. A silicon wafer is a flat, well-defined, contamination-free, and affordable substrate. The mold comprises circular holes several tens of nanometers deep as the basic feature; these holes will be reproduced as nanopillars in the hydrogel. We have fabricated a set of samples with varied sputtering parameters (ion dose, beam current, and milling time) to assess the suitability of the mold. Based on these tests, we have selected two functional stamp molds. The first one has an area 540 × 540 µm^2^ and comprises 1,361,700 features milled using a 160 pA beam current (fabrication time 15 h). The second mold features two arrays of 90 × 90 µm^2^ each consisting of 37,825 features of distinct feature sizes fabricated using beam currents of 42 pA and 160 pA, respectively, with a total fabrication time of 1 h. The smaller beam current typically entails smaller milled dimensions of the features; therefore, for simplicity, we denote 42 pA as *small* and 160 pA as *big* features. The overview and detailed images acquired by SEM (5 kV, 160 pA) of molds are shown in Figs. 1a, b, and 2. An arrow-shaped marker (150 × 150 µm^2^) enabled easy optical localization of the patterns.

### Hydrogel PHEMA preparation

**Figure 3:**
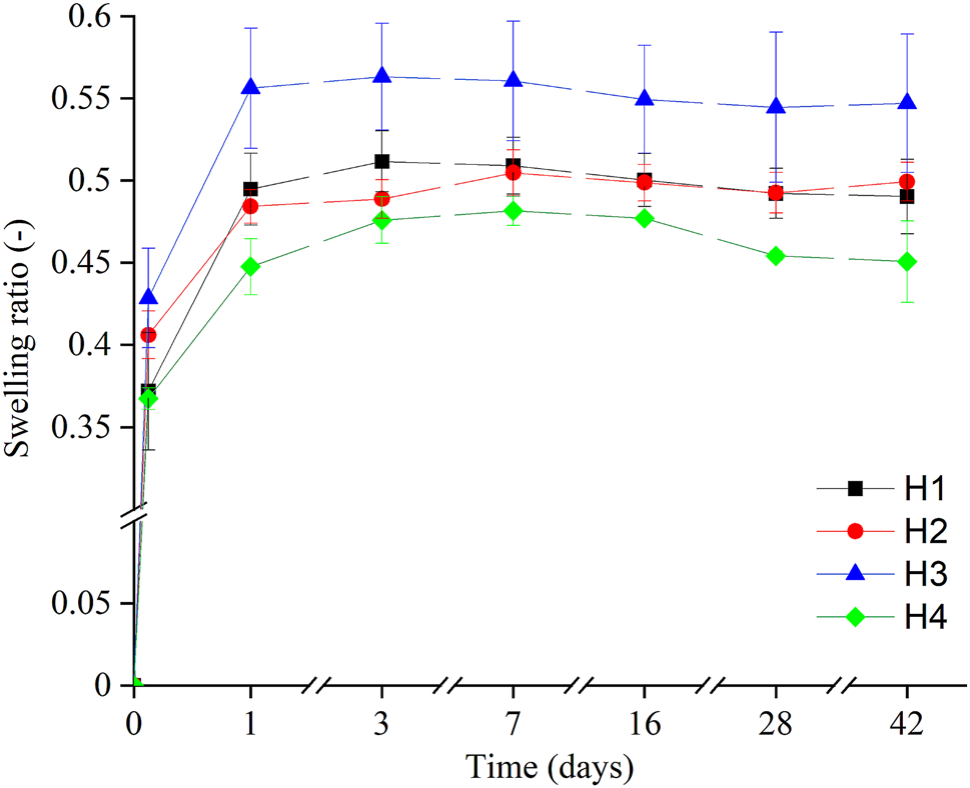
Swelling ratio of hydrogel blends H1 (black), H2 (red), H3 (blue), and H4 (green). Measured points are connected by auxiliary lines to guide the eye.

A critical point for mold lithography is using a hydrogel with properties that guarantee sufficient swelling, hydrolytic stability, and, more importantly, facile hydrogel peeling from the Si mold without damaging both the hydrogel and the mold. To obtain the required behavior, the effect of composition was investigated by preparing HEMA blends in different weight ratios (Table 1). Four compositions of the PHEMA hydrogels (H1, H2, H3, and H4) with distinct concentrations of EGDMA and HEMA were prepared, analyzed, and tested. The dependence of the swelling ratio of each hydrogel is presented in Fig. 3. The smaller amount (0.25 wt.%) of EGDMA in the H3 blend leads to a lower density of the polymer network and, consequently, to higher water absorption and an inability of the hydrogel to maintain its original shape and water content. On the contrary, the H4 blend had a higher concentration (1.20 wt.%) of EGDMA, leading to a lower swelling due to the higher density of the polymer network. However, the hydrogel was unable to maintain water equilibrium: the water retention showed a decreasing trend (water content decreased by 3% between 7 and 42 days), unsuitable for long-term application for IOL. Blends H1 and H2 with 0.85 wt% of EGDMA reach sufficient hydrogel swelling and stability after three days (H1) and seven days (H2) with the swelling ratio up to 0.5 compared to the original weight.

**Table 1:** Composition of PHEMA hydrogels.

	HEMA monomer (wt.%)	Water (wt.%)	EGDMA (wt.%)	DMPA (wt.%)
H1	92.47	5.50	0.85	1.18
H2	60.00	37.97	0.85	1.18
H3	92.47	6.10	0.25	1.18
H4	92.47	5.15	1.20	1.18

Both blends were crosslinked in the mold, swelled in ultrapure water, and peeled off. We found that H2 does not form a high-quality nanostructured surface and, in addition, PHEMA residues remain on the mold surface. H2 hydrogel is softer than H1 due to higher water content in the polymer mixture; as a result, the prepared nanostructures did not keep their shape. The mold was cleaned from H2 residues by pouring it into a mixture of water and ethanol^25^ for a few hours. Nanostructures fabricated from the H1 blend were relatively rigid, and no residues were left on the mold; hence, all the following experiments were performed on the H1 blend.

### PHEMA nanostructuring

The nanostructured PHEMA surface was fabricated using a silicon mold with a lithographic pattern. First, the DMPA photoinitiator with a given concentration (see Table 1) was added to the HEMA monomer. Then the crosslinking agent EGDMA and ultrapure water were added; the resulting mixture was stirred until a homogeneous liquid was obtained. The solution was then poured onto the Si mold and irradiated with UV radiation (wavelength 365 nm, power 36 W) for 10 min under ambient conditions using a UV lamp placed 15 cm from the sample. The wavelength was chosen based on the high absorption of the radiation by DMPA at 365 nm^26^; our experimental data show that the proper polymer crosslinking of hydrogel was achieved after 10 min of irradiation. After the irradiation, the PHEMA hydrogel was immersed in ultrapure water to remove unreacted residues; the hydrogel was removed from the water environment and its surface gently cleaned with filtration paper. Afterwards, the hydrogel was immersed in fresh ultrapure water for another two days to remove the remaining unreacted components. When the PHEMA hydrogel was not used for the experiment, it was stored in the water environment; after each experiment, the water was replaced with a fresh load.

### Characterization of the nanostructures on PHEMA and stamp mold

**Figure 4:**
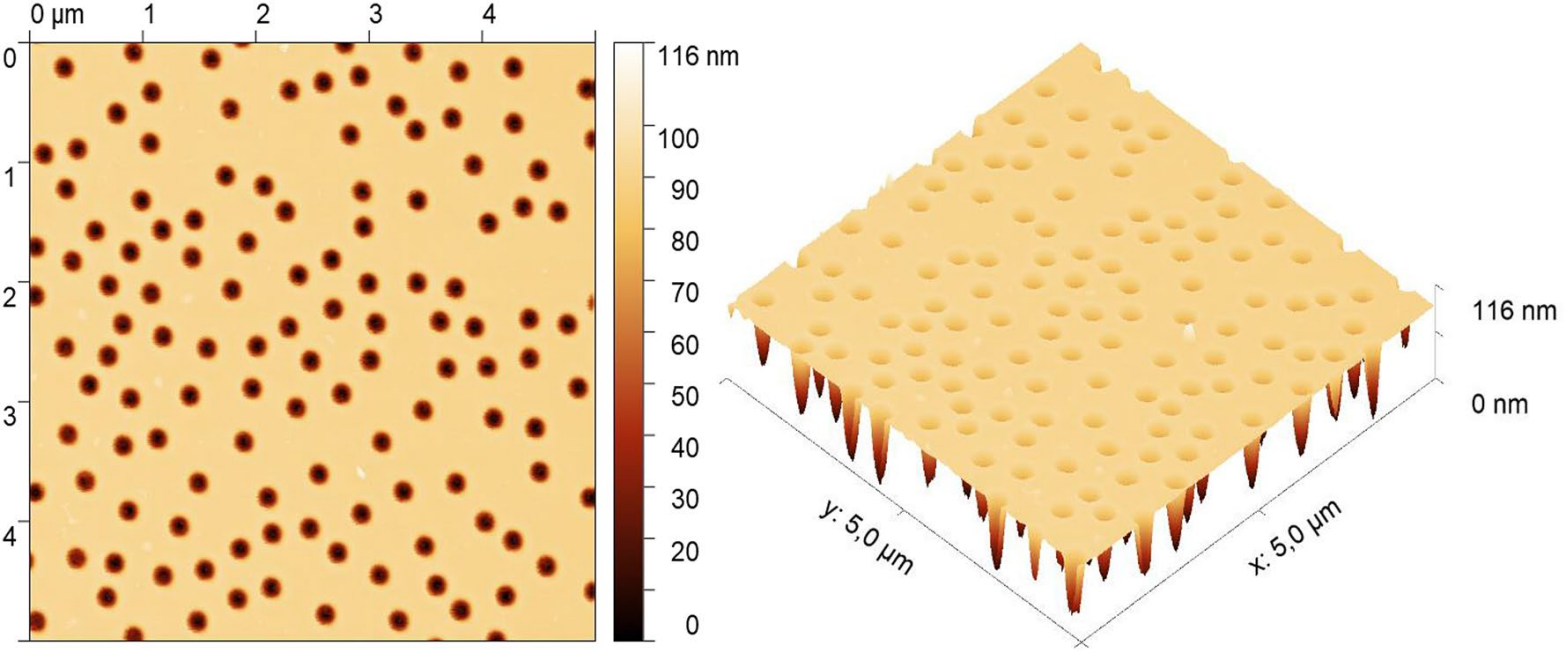
Detailed AFM image of silicon mold with the array consisting of big holes.

**Figure 5:**
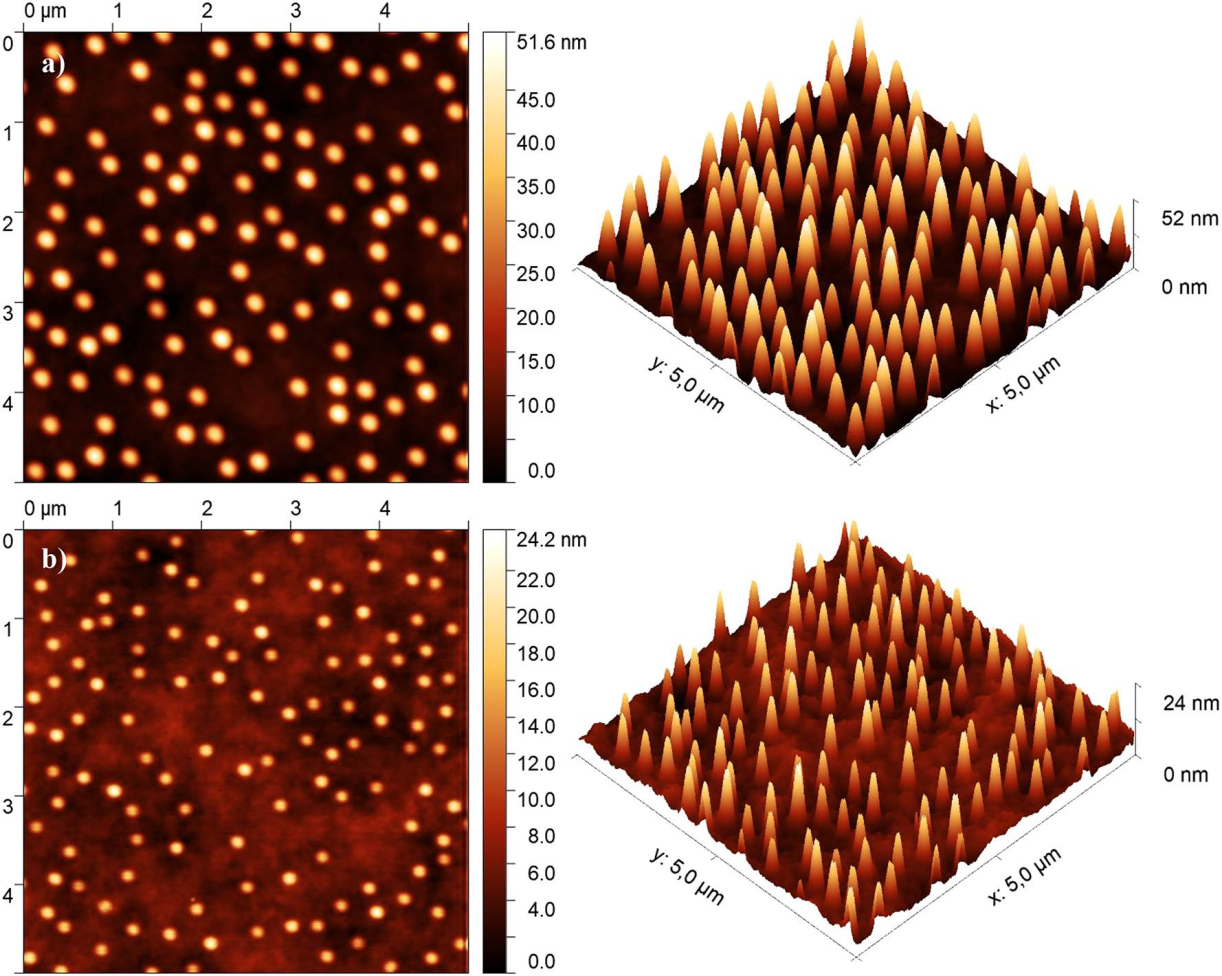
AFM images of the (**a**) *big* and (**b**) *small* imprinted nanopillars on the hydrogel.

We have characterized the fabricated molds and pristine and nanostructured PHEMA surfaces employing a set of analytical methods. The sizes of the holes in the mold and nanopillars on the hydrogel were determined by AFM. The measured silicon mold with a sputtered area of 540 × 540 µm^2^ consisting of *big* holes is shown in Fig. 4, and the arrays of *big* and *small* nanopillars on hydrogel samples can be seen in Fig. 5. When the hydrogel was taken from a liquid environment, it immediately started to shrink; therefore, it had to be partially dried to reach a stable composition and size, which allowed the AFM measurements without significant drift. The drying process with the AFM measurement took two hours in total. Another requirement for the AFM is a planar surface. For the reference sample measurement, the planar surface was obtained by shaping the hydrogel blend employing the plastic rectangular box as a mold.

All measured nanopillars were approximately circular; therefore, their diameter could be calculated from their area using the equation for the area of the circle. The nanopillar areas were determined using the thresholding procedure implemented in the ImageJ software; threshold was set to 89% and 94% of the total height for big and small nanopillars. The parameters of individual pillars/holes were statistically processed at a reliability level of 95%. We note that the top of the hole and bottom of the pillar are significantly broader than the rest of the nanopillar as a result of the presence of significant broadening of the FIB beam profile by low intensity exponential tail^27,28^; hence, this procedure should be performed carefully. The hole depth in the silicon mold and the imprinted nanopillar height obtained from the AFM data are shown in Tables 2 and 3. The depths of both the big and small holes in the stamp mold were consistent and matched the calculated values, as shown in Table 2. The theoretical calculations are described in the Supplementary Information.

**Table 2:** Dimensions of the *big* and *small* nanopillars milled in the stamp mold (data reliability 95%) with theoretically calculated depth lengths.

Stamp mold	Beam current	Measured		Calculated
		Diameter (nm)	Depth (nm)	Depth (nm)
	42 pA	168 ± 23	21.5 ± 2.5	18.3
	160 pA	238 ± 20	84 ± 3	69.8

**Table 3:** Influence of hydrogel drying on the dimensions of the nanopillars (data reliability 95%).

Hydrogel	Inviolability	Time in air	Beam current	Measured	
				Height (nm)	Diameter (nm)
	Fresh	2 h	42 pA	16 ± 5	160 ± 30
			160 pA	43 ± 6	244 ± 27
	Used	2 h + 2 h	42 pA	13.2 ± 1.6	152 ± 20
			160 pA	31 ± 3	215 ± 17

Table 3 shows the AFM results of the measured dimensions of the nanopillars on the freshly prepared hydrogel sample (labeled as *fresh*) and the results of the same sample measured after four days, during which the sample was stored in distilled water to prevent it from drying (labeled as *used*). The used sample was also partially dried before each AFM measurement, as described above.

### Chemical characterization of the nanostructured and unmodified PHEMA

**Figure 6:**
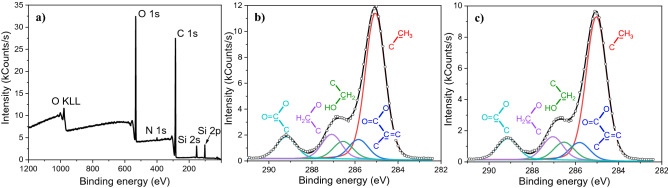
Photoelectron spectra of the dried PHEMA surface with shown components in the (**a**) survey spectrum and the detailed C 1s spectra of (**b**) unmodified and (**c**) modified surface with fitted components.

The chemical analysis of dried PHEMA was performed using XPS and FTIR. The photoelectron spectra of PHEMA samples show the carbon C 1s and oxygen O 1s peaks expected for PHEMA; in addition, small peaks of nitrogen N 1s and silicon Si 2p and 2s are present (Fig. 6a). The silicon peaks probably originate from the silicon stamp mold and N 1s from the environment during hydrogel treatment. We focused on the C 1s as it is the most informative for the chemical characterization of HEMA-based polymers. We determined five peak components in the C 1s peak, referring to typical groups presented in PHEMA^23^^,^^29–32^. We compared the photoelectron spectra measured on the unmodified (Fig. 6b) and nanopatterned surface (Fig. 6c). As there were no observable differences, we concluded that the modification of the PHEMA hydrogel has no measurable influence on the resultant chemical composition.

**Figure 7:**
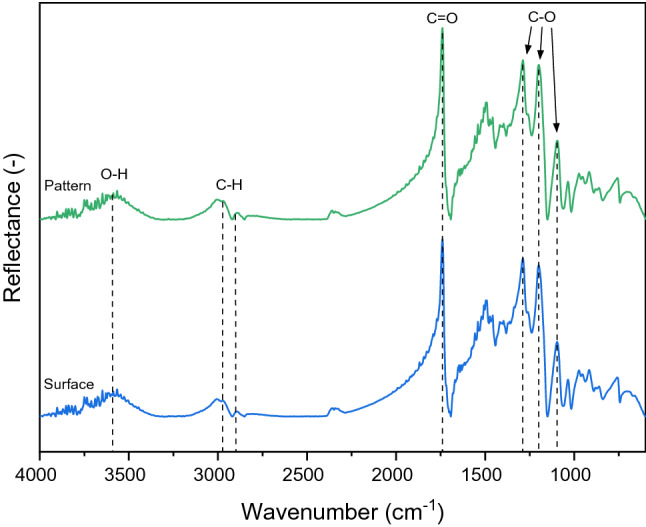
FTIR spectra of the dried hydrogel PHEMA samples on the unmodified (Surface) and nanopatterned surface (Pattern).

The FTIR analysis was employed to identify chemical bonds within PHEMA samples. The FTIR spectra of the unmodified and patterned hydrogel surface are shown in Fig. 7. We determined characteristic methylene, carbonyl, and hydroxyl groups commonly presented in PHEMA hydrogels^33^. The spectra comprised –OH stretching vibration bands at 3590 cm^–1^ with a lower intensity and a slight band shift toward the higher frequencies caused by PHEMA dehydration before the measurement^34^. Stretching vibrations of C–H appeared around 3975 and 2890 cm^−1^; the stretching of C=O was observed at 1740 cm^−1^, and the absorption band of C–O characteristic for PHEMA was observed with peaks at 1290 cm^−1^, 1197 cm^−1^, and 1094 cm^–1^. All recorded data in the spectra confirmed that the chemical composition on the patterned and unmodified surface is identical.

### Wide-angle X-ray scattering (WAXS) of the PHEMA hydrogels

**Figure 8:**
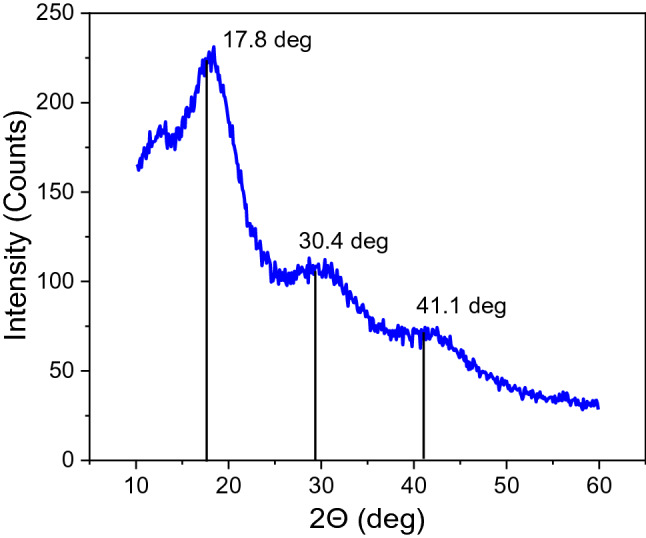
Radial intensity profile obtained from WAXS measurement of the dried PHEMA hydrogel sample. The peak positions are marked by vertical lines.

We used X-ray scattering (WAXS) to study the atomic structure of the dried non-crystalline PHEMA samples. In the transmission mode, we scanned over the whole sample to see any change in the spectra associated with the nanopattern. We identified isotropic Debye rings in two-dimensional diffractograms (see Supplementary Information) and reduced them into radial profiles by azimuthal integration of the intensity. In the spectra (Fig. 8), we observed three broad peaks located at 17.8°, 30.4°, and 41.1°, corresponding to d-spacings (0.50 ± 0.03) nm, (0.29 ± 0.01) nm, and (0.22 ± 0.01) nm, respectively. The first two peaks are similar to the results with PHEMA material^35^, where the first peak was attributed to the polymer, while the second was attributed to the pair distribution of water molecules.

### Biocompatibility and light transmissivity

**Figure 9:**
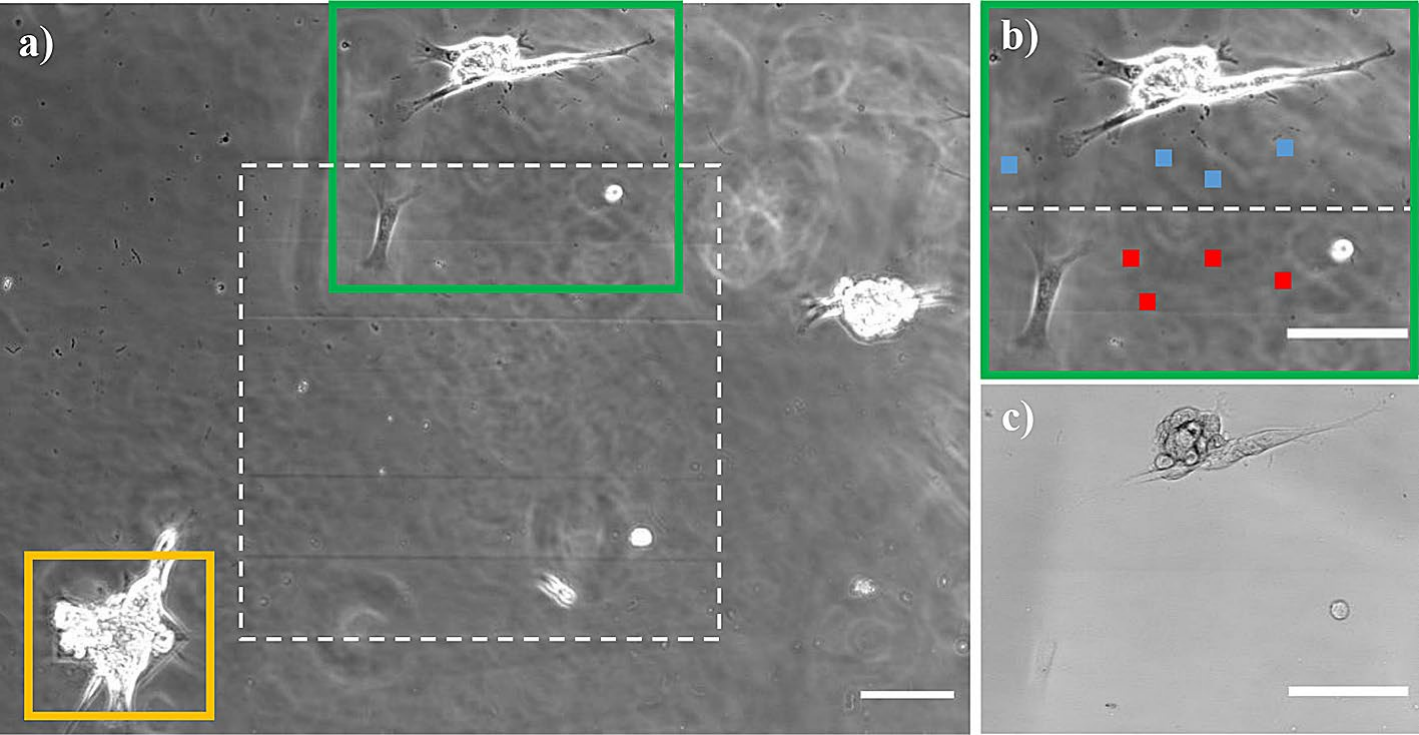
Hydrogel surface with cultured human fibroblasts (scale bars 100 µm). Phase-contrast image (**a**) shows the surface with the nanopillar array (white rectangle); the sputtered arrow shape (see Fig. 1) is covered by the cell marked by the yellow rectangle. Image (**b**) shows the detail of the area (green rectangle) consisting of unmodified hydrogel surface (top half) and nanopattern (bottom half) with the corresponding bright-field image given in (**c**).

To assess the biocompatibility, we cultured human fibroblasts on the PHEMA hydrogel surface (three cells shown in Fig. 9a). The fibroblasts did not show any signs that would hint at the presence of substrate cytotoxicity during the four-day observation through the microscope. The fluorescence image of the fibroblast is shown in Supplementary Information.

To evaluate the light transmissivity, we focused on the surface of the nanopatterned hydrogel and measured the average light intensity in four regions of interest (32 × 32 pixels) within the nanostructured area and four outside (red and blue marks in Fig. 9b). The mean greyscale intensity was 6851 ± 46 (1 S.D.) for the nanostructured surface and 6786 ± 72 for the reference surface: there was no significant difference between the measurements on the nanopatterned area and outside (t-test P value = 0.17). The possible small increase would be in line with the theoretical concepts where oblique parts of surfaces reduce reflections and therefore increase transmissivity^36^. Hence, we concluded that the introduced nanopattern does not influence the transmissivity of the PHEMA hydrogel. Additionally, the nanopillars are not visible in the bright field or even in phase-contrast images (acquired with Plan Fluor 10 × NA 0.3 Ph1 objective lens) as expected (Fig. 9c).

## Discussion

The repeatability of the stamp mold process was checked by preparing ten patterned hydrogel samples, and the resultant patterned arrays were still the same. The crucial factor for good repeatability is to have well-balanced components in the hydrogel blend that do not leave residues on the mold surface. We determined the best component ratio (labeled H1 in Table 1) as 92.47 wt.% for the HEMA monomer, 5.50 wt.% for ultrapure water, 0.85 wt.% for EGDMA, and 1.18 wt.% for DMPA. The other considered hydrogels H2–H4 were unsuitable for our application for various reasons, including the inability to maintain the original shape, formation of imperfect nanopillars on the surface, or inability to keep the water volume in time.

The diameter of the hydrogel nanopillars stays almost the same for the *fresh* (*small* 95%, *big* 102% of the actual holes' diameter) and the *used* sample (*small* 90%, *big* 90% of the actual holes' diameter). The measured hole depths in the mold correspond to theoretically obtained values (the difference is about 16%). On contrary, the height for the freshly made *small* and the *big* hydrogel nanopillars is 74% and 49% of the actual depth of the holes in the mold, probably because the hydrogel blend does not fill the mold holes entirely. Moreover, *fresh* hydrogel loses water under ambient conditions and shrinks in size. Hence, the *used* sample exhibited a decrease of about 22.5% in height and 8.5% in diameter of the nanopillars compared to the *fresh* sample. Additionally, the size reduction is partially irreversible as the *used* hydrogel could not fully recover in water to its previous size.

We have evaluated and compared the mechanical and chemical properties of the nanopatterned hydrogel PHEMA with the unmodified one. We did not find any reduction in light transmissivity, transparency, or reduction of biocompatibility. Hence, nanopatterning has no detrimental effect on ophthalmic applications. Further experimental work will be required to evaluate the effect of the nanopattern on the cells cultured on its surface.

## Conclusions

To summarize, we have fabricated an array of nanopillars on the hydrogel surface employing the FIB fabricated silicon mold for the imprint. We have used the molds to produce a hydrogel surface featuring 1,361,700 nanopillars randomly positioned within the area of 540 × 540 μm^2^, which is sufficient for cell attachment applications. AFM imaging shows that the hydrogel nanopillars are of superior quality and without defects. The nanopillars display a narrow size distribution both in height and diameter. Their nano-dimensions ensured their full transparency.

We determined the chemical composition and presence of bonds of the unmodified and nanopatterned PHEMA by XPS and FTIR. These measurements confirmed that hydrogel properties are retained during the preparation. The chosen hydrogel PHEMA is a common biocompatible and nontoxic hydrogel material, as demonstrated by our biocompatibility tests.

The array can comprise millions of randomly positioned or long-range-ordered nanostructures with well-defined shapes. The nanostructures on hydrogel surfaces can find a broad range of applications in tissue engineering, drug delivery, ophthalmology, and fundamental cell biology research. Fast preparation and reproducibility of the nanostructured hydrogels could be attractive for the biomedicine industry, e.g., cataract-resistant nanostructured intraocular lenses. The biological efficacy of nanopatterned PHEMA hydrogels against cytoskeleton protein fouling is being evaluated in vitro on human dermal fibroblasts and will be the subject of future research.

## Supplementary Information

Supplementary Figures.

Supplementary Information.

## Abbreviations

IOL: Intraocular lens
HEMA: 2-Hydroxyethyl methacrylate
PHEMA: Poly(2-hydroxyethyl methacrylate)
EGDMA: Ethylene glycol dimethacrylate
DMPA: 2,2-Dimethoxy-2-phenyl acetophenone
UV: Ultraviolet
FIB: Focused ion beam
SEM: Scanning electron microscopy
AFM: Atomic force microscopy
XPS: X-ray photoelectron spectroscopy
FTIR: Fourier-transform infrared spectroscopy
WAXS: Wide-angle X-ray scattering
