# Supplementary figures and images for: Replica-mold nanopatterned PHEMA hydrogel surfaces for ophthalmic applications
Source: Sci Rep. 2022 Aug 25;12:14497. doi: 10.1038/s41598-022-18564-3 (PMC9411613; doi:10.1038/s41598-022-18564-3)

## Slide 1
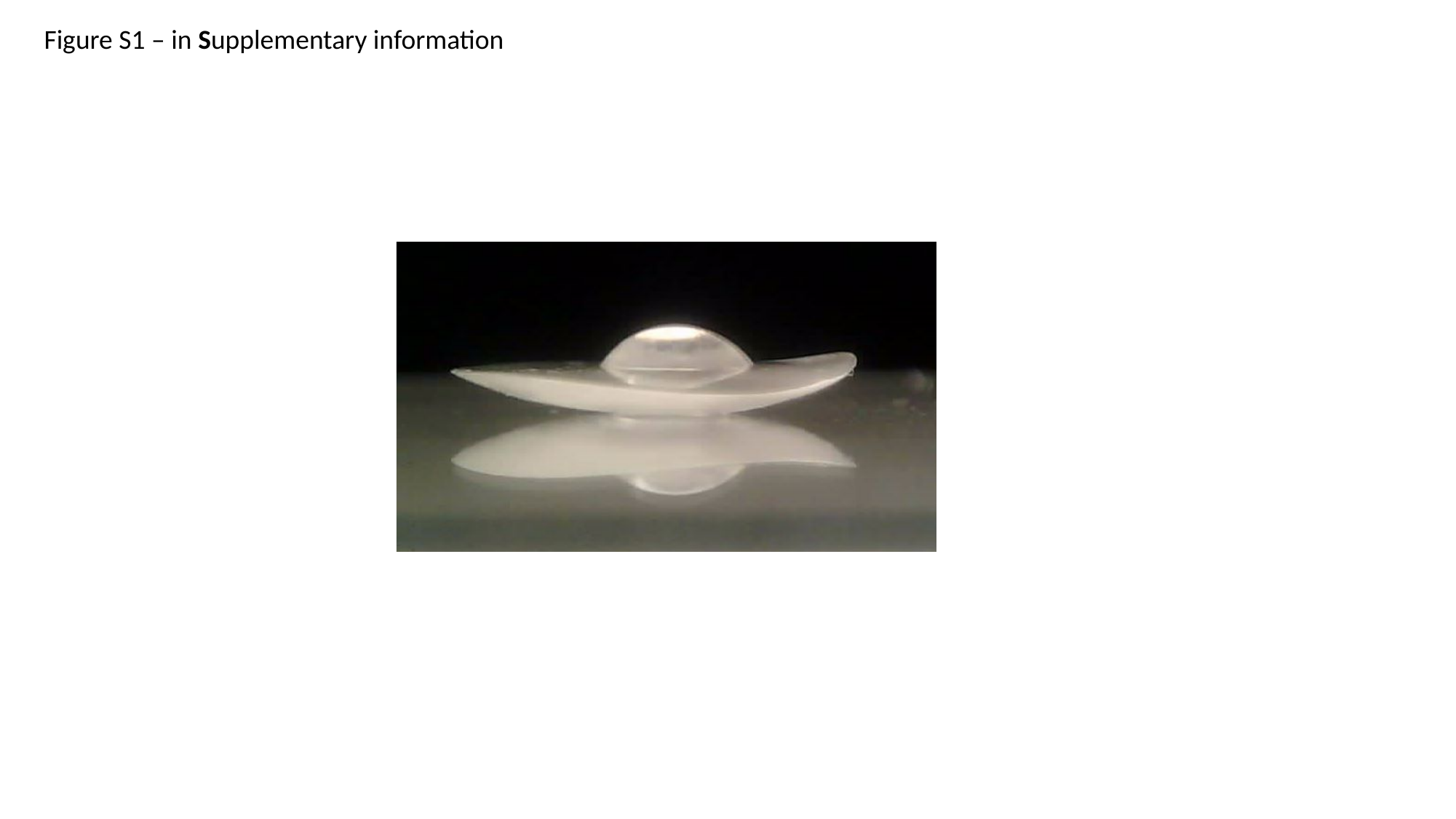

Figure S1 – in Supplementary information

## Slide 2
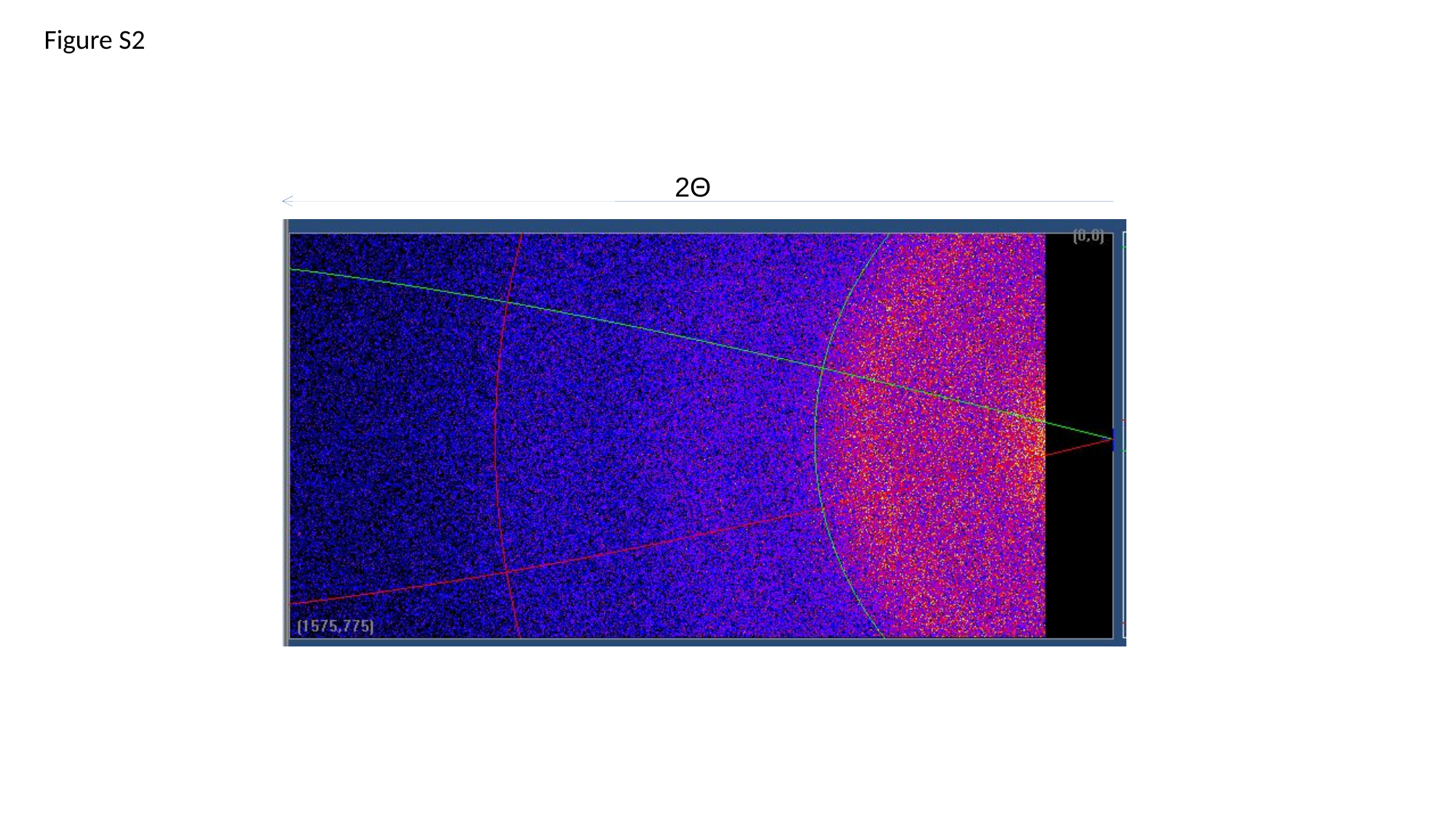

Figure S2
2Θ

## Slide 3
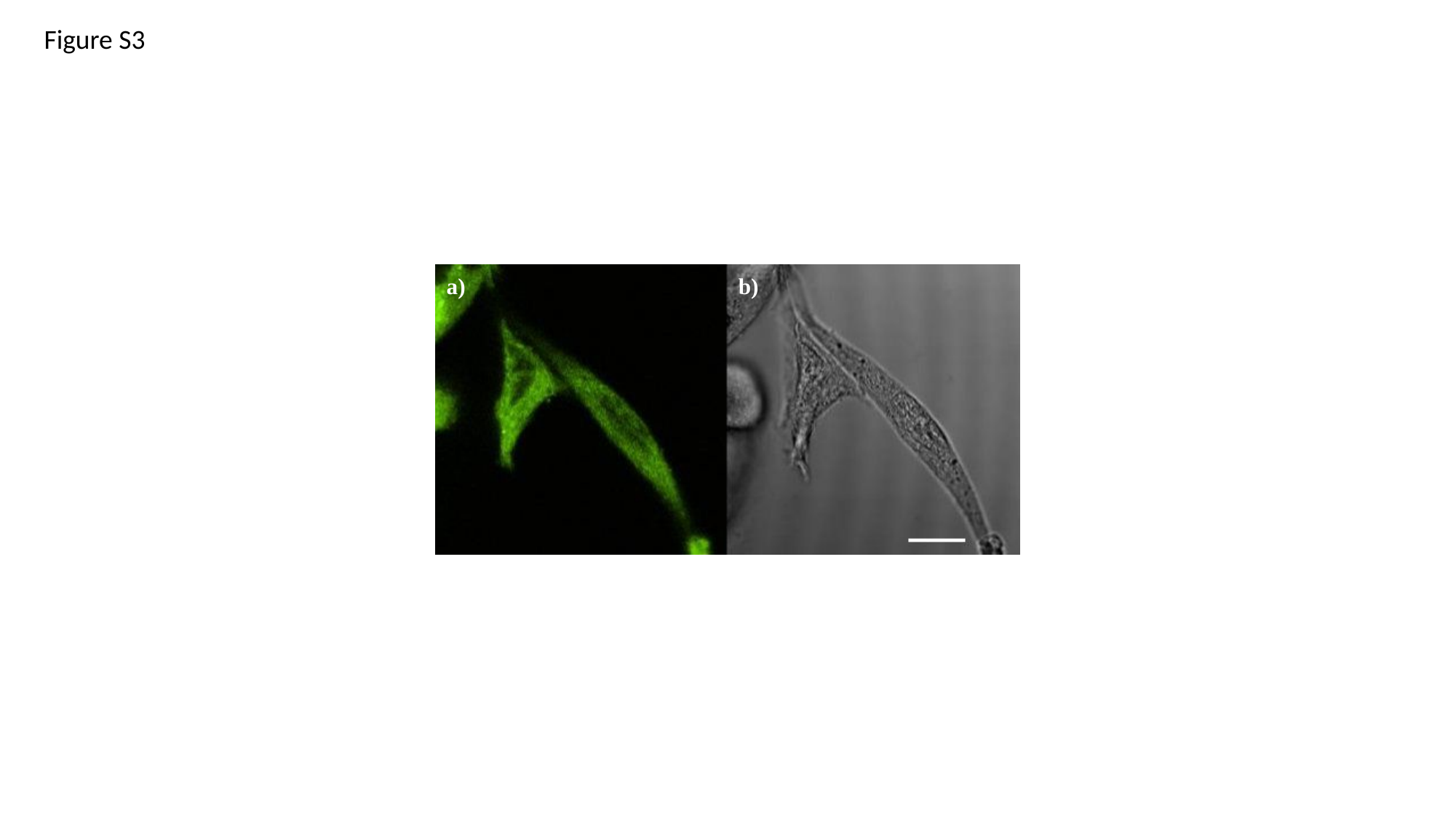

Figure S3
b)
a)

Supplement: Supplementary file 1 — Supplementary Figures. [file 41598_2022_18564_MOESM1_ESM.pptx]
