# Supplementary material for: Replica-mold nanopatterned PHEMA hydrogel surfaces for ophthalmic applications
Source: Sci Rep. 2022 Aug 25;12:14497. doi: 10.1038/s41598-022-18564-3 (PMC9411613; doi:10.1038/s41598-022-18564-3)
Supplement: Supplementary file 2 — Supplementary Information. [file 41598_2022_18564_MOESM2_ESM.docx]

**SUPPLEMENTARY INFORMATION**

**Replica-mold nanopatterned PHEMA hydrogel surfaces for ophthalmic applications**

Tomáš Krajňák^1,*^, Eva Černá^1^, Markéta Šuráňová^2^, Tomáš Šamořil^1,2^, Daniel Zicha^1,2^, Lucy Vojtová^1,*^, Jan Čechal^1,2^

^1^ CEITEC – Central European Institute of Technology, Brno University of Technology, Purkyňova 123, 612 00 Brno, Czech Republic.

^2^ Institute of Physical Engineering, Brno University of Technology, Technická 2896/2, 616 69 Brno, Czech Republic. 

*** Corresponding Authors:** Tomáš Krajňák, Lucy Vojtová
Tel.: +420 54114 9832 (L.V.)
E-mail: tomas.krajnak@ceitec.vutbr.cz (T.K.), lucy.vojtova@ceitec.vutbr.cz (L.V.)

**SCRIPT**

A script to generate an array of nanopillars was written in Python. The main idea was to generate a square area composed of randomly distributed points as a template; multiples of this basic area were placed to a 6 × 6 array. The dimensions of the original template were 90 × 90 µm^2^ with 37,825 points. The area of the final structure was then 540 × 540 µm^2^ with 1,361,700 points.

The numbers in the script were chosen in nanometer units to have a fine mesh grid. The *Randint* function from the Python random package [1] was used to achieve the random distribution of the points. Two variables [xPos, yPos] were generated, representing the point's x and y positions. The [xPos, yPos] were stored in the vector called Points. Then the new [xPos, yPos] were generated within the *while* cycle and compared with the stored variables in Points. If the new point did not lie within the Euclidean distance [2] (set to 300 nm), the new point was added. The cycle ended after filling the vector Points by 37,825 points. The diameter of the points was determined by beam current during fabrication; therefore, it was not specified in the script.

The calculation speed of the process needed to be optimized to avoid software memory being filled with all data. Therefore, all the positions of the points in the Points file were saved into a .txt file in the format compatible with the lithography software. Then each point from Points was shifted by 90 µm in x, then in y, and finally in both axes. The resultant array is 2 × 2, with the origin in the top left corner. The shifted positions of the new points were saved in the same .txt file as previously. This approach allows for the acquisition of 3 × 3 and higher rank structures by changing the value of the point at the end of the current rank structure and just writing the values into.txt without storing them in software memory. This approach provides a good calculation speed for a large number of values.

The final .txt file was then imported into readable .xml format for the LYRA3 FIB-SEM lithography software module. The user needs to adjust the FIB and set the sputtering parameters such as depth (ion dose), beam current, and array position.

**Water contact angle**

The water contact angle determines surface wettability, which is a significant property toward the final application. The hydrated PHEMA hydrogel is naturally hydrophilic: the applied water droplet quickly spreads over the hydrogel surface. On the surface of dried hydrogel, the measurement had to be carried out quickly because hydrogel absorbs the applied water. The measured contact angle on dried hydrogel was (51 ± 5) °, indicating the hydrogel's hydrophilic behavior.


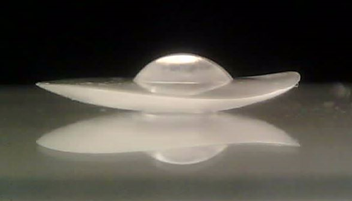

**Figure S1:** Water contact angle measured on PHEMA hydrogel.

**WIDE-ANGLE X-RAY SCATTERING (WAXS)**

Two-dimensional diffractogram from WAXS measurement of the dried PHEMA sample is shown in Figure S2.


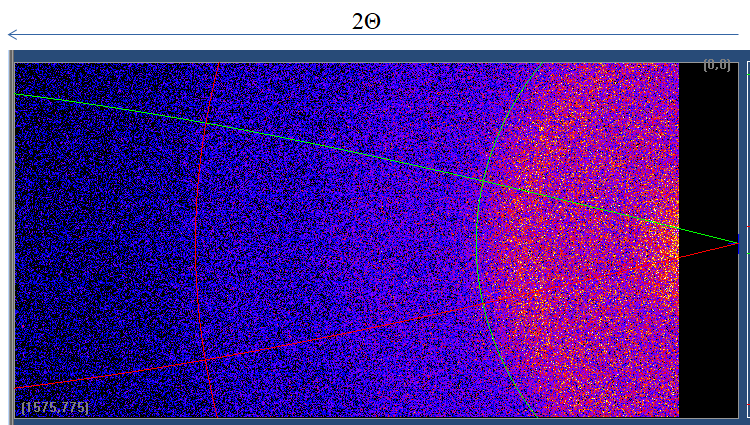
**Figure S2:** Two dimensional diffractogram of the PHEMA sample.

**Fluorescence**

The cell morphology of human fibroblast cultivated on PHEMA surface was measured by confocal microscope in fluorescence mode. The fluorescence and its corresponding optical images are shown in Figure S3a and S3b.

**Figure S3:** a) Fluorescence and b) optical images of the human fibroblast cell on PHEMA surface (scale bar 10 µm).


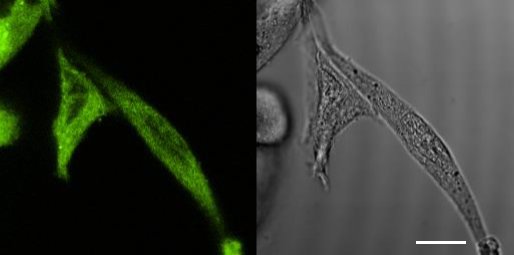


**a)**

**b)**

**THEORETICAL CALCULATIONS**

The sputtered depth by FIB can be determined by ion dose, which is the number of ions related to unit of area [3, 4]. Ion dose *D* can be calculated by equation (1):

$D = \frac{ItN}{eb^{2}}$, (1)

where *I* is the ion beam current, *t* is dwell time in the point, *N* is the number of scans, *e* is the elementary charge, *b* is the spot size. If the sputtering rate *R* for a specific material is known, the depth *h* is defined by equation (2):

$h=DeR$. (2)

The used sputtering rate for silicon in the calculations was *R* = 0.3 µm^2^.nA^-1^.s^-1^ (Ga ions, 30 keV) defined in the FIB instrument.

**LITERATURE:**

[1] Van Rossum, G. The Python Library Reference, release 3.8.2. Python Software Foundation (2020).

[2] Liberti, L. & Lavor, C. Euclidean Distance Geometry. *Springer International Publishing* (2017). doi:10.1007/978-3-319-60792-4.

[3] Bischoff, L. & Teichert, J. Focused Ion Beam Sputtering of Silicon and Related Materials. Forschungszentrum Rossendorf; FZR-217 (1998).

[4] Ali, M. Y., Hung, W. & Yongqi, F. A review of focused ion beam sputtering. Int. J. Precis. Eng. Manuf. 11, 157–170 (2010).
